# Anemia and Its Determinants among Male and Female Adolescents in Southern Ethiopia: A Comparative Cross-Sectional Study

**DOI:** 10.1155/2020/3906129

**Published:** 2020-10-09

**Authors:** Melat Belay Zeleke, Mohammed Feyisso Shaka, Adane Tesfaye Anbesse, Solomon Hailemariam Tesfaye

**Affiliations:** School of Public Health, College of Health Sciences and Medicine, Dilla University, Dilla, Ethiopia

## Abstract

**Background:**

Adolescent anemia is a major public health problem worldwide. Adolescents (10–19 years) are at an increased risk of developing anemia due to increased iron demand during puberty, menstrual losses, limited dietary iron intake, and faulty dietary habits.

**Objective:**

To assess the prevalence of anemia and associated factors among male and female adolescent students in Dilla Town, Gedeo Zone, Southern Ethiopia, May 2018.

**Methods:**

A school-based comparative cross-sectional study was employed among 742 school adolescents. Basic characteristics, anthropometric measurements, haemoglobin measurement, and others were collected. Data were analyzed using SPSS version 20 software, and descriptive statistics were computed for all variables. Bivariate and multivariable logistic regression analyses using binary logistic regression were done, the results were interpreted by using AOR with their corresponding 95% CI, and statistically significant difference was declared at *p* < 0.05

**Result:**

Out of the total 742 respondents, 377 (50.8%) were males and 365 (49.2%) were females. The overall prevalence of anemia was 21.1%, and the prevalence of anemia was 22.5% among male adolescents and 19.7% among females. Male adolescent students within the early adolescence age group (10–13 yrs) (AOR 0.27, 95% CI, 0.08–0.87), those consuming fibre-rich foods daily (AOR 0.11, 95% CI, 0.02–0.61), and those having no intestinal parasites (AOR 0.04, 95% CI, 0.02–0.09) were less likely to be anemic. Similarly, female adolescent students not having intestinal parasites (AOR 0.05, 95% CI, 0.01–0.11) were less likely to develop anemia while those from malaria endemic area (AOR 2.57, 95% CI, 1.13–5.83) were identified to be more anemic.

**Conclusion:**

This study identified that anemia was a moderate public health significance in the study area, and the prevalence of anemia was slightly higher among male than female adolescents. Age category, frequency of eating fibre-rich foods, and positive intestinal parasite tests were factors contributing for anemia among male adolescents while presence of intestinal parasite and malaria endemicity were the determinants of anemia among female adolescents.

## 1. Background

Anemia is defined as a decrease in the concentration of circulating red blood cells or haemoglobin concentration resulting in insufficient oxygen carrying capacity of red blood cells to meet the physiological needs of the body [1, 2]. Anemia generally results from blood loss, decreased red blood cell (RBC) production, poor RBC maturation, or increased RBC destruction [3, 4]. The WHO defines anemia in adolescents as a haemoglobin value below 11 g/dl for children 5–11 years of age, below 12 g/dl for children 12–14 years of age, below 13 g/dl in men 15 years of age, and above and below 12 g/dl in nonpregnant women 15 years of age and above [2]. Preschool children, pregnant woman, and adolescents constitute a vulnerable group for anemia [5].

According to the World Health Organization (WHO), adolescence has been defined as the period of life between 10 and 19 years of age [6] and constitutes about 25% of the world population. During adolescence, the velocity of growth is at the faster stage next to the critical window of the first 1,000 days of life, and adequate nutrition during this age is very important than any other age. This is the formative period of life when the maximum amount of physical, psychological, and behavioural changes takes place [7].

During childhood and adolescence period, the nutritional needs of boys slightly differ from that of girls. Iron requirements peak during adolescence due to rapid growth and increase in blood volume. Anemia during adolescence causes reduced physical and mental capacity and diminished concentration in work and educational performance, and also poses a major threat to future safe motherhood in girls [8]. Despite the fact that iron is the second most abundant metal in the earth's crust, anemia is considered as the most common micronutrient deficiency worldwide, and in 95% of cases, it is associated with an iron-poor diet. Iron deficiency is thought to be the most common cause of anemia [9, 10].

Globally anemia affects the lives of more than 2 billion people, accounting for over 30% of the world's population which is the most common public health problem particularly in developing countries occurring at all stages of the life cycle [11]. In developed countries, 4.3 to 20% of the population, depending on age and gender, are affected by iron deficiency anemia, while in developing countries, these figures range from 30 to 48% [3]. The worldwide prevalence of anemia among adolescents is 15% (27% in developing countries and 6% in developed countries) [12]. Notwithstanding this, few data are currently available on the prevalence of iron deficiency anemia among adolescents.

Adolescents are at an increased risk of developing anemia due to increased iron demand during puberty, menstrual losses, limited dietary iron intake, and faulty dietary habits. This dramatic increase in iron requirement among adolescents peaks between the ages of 14-15 years for girls and one to two years later for boys. The requirement for iron doubles during adolescence as compared to the younger age group. The overall iron requirement increases two to three folds from a preadolescence level of approximately 0.7–0.9 mg Fe/day to as much as 1.37–1.88 mg Fe/day in adolescent boys and 1.40–3.27 mg Fe/day in adolescent girls. This is due to the expansion of total blood volume, increase in lean body mass, and the onset of menstruation in adolescent females [13, 14].

In spite of increased iron needs, many adolescents, particularly females have iron intakes of only 10-11 mg/day of total iron, resulting in approximately 1 mg of absorbed. About three fourths of adolescent females and 17% of males do not get their dietary iron requirement which makes the adolescents vulnerable to the development of anemia [15]. The most common causes of anemia in adolescent, especially in developing countries and Sub-Saharan Africa, are poverty and ignorance that leads to lack of purchasing power to afford foods containing heme iron, low socioeconomic status leader to poor sanitation and hygiene, low iron intake, poor bioavailability of dietary iron, infections, and parasitic infestation [16].

Anemia in adolescent has serious implications for a wide range of outcomes, and nearly all of the functional consequences of iron deficiency are strongly related to the severity of anemia. It causes reduced resistance to infection, impaired physical growth and mental development, and reduced physical fitness, work capacity, and school performance [17, 18].

In Ethiopia, adolescents, who constitute a sizable segment of its population, form a vulnerable group and are at a greater risk of morbidity and mortality. Anemia is widely prevalent in Ethiopia and affects both sexes in all age groups. Among adolescents, girls constitute a vulnerable group, particularly in developing countries such as Ethiopia. In a family with limited resources, the female child is more likely to be neglected due to sociocultural factors [19]. In Ethiopia, the prevalence of anemia among the age group of 15–19 year-old males and females was 18.2% and 19.9%, respectively [20].

Even though the study of anemia among both male and female adolescents is very limited in Ethiopia, few studies have been conducted on school female children, reproductive age women, and pregnant women and the results of all these studies put anemia as a moderate-to-severe public concern. The National Nutrition Program (NNP) of Ethiopia gives special attention to address these age groups, but the implementation of this program at the grass root level is very weak as compared with other vulnerable groups [21].

Therefore, the main aim of this study was to compare the prevalence of anemia and associated factors of anemia among male and female school adolescents in Dilla Town. Results from this research are important to clarify how to approach with adolescent-specific nutrition intervention to reduce anemia-related morbidity and increase productivity. Also, the findings will be used as input for different health sectors at zonal and woreda levels to plan interventions to reduce the problem of anemia.

## 2. Methods and Materials

### 2.1. Study Design and Setup

A school-based comparative cross-sectional study was conducted from 14^th^ May to 1^st^ June 2018 among school adolescents (10 to 19 years) in Dilla Town, Gedeo Zone, Southern Nations, Nationalities, and Peoples' Region (SNNPR), Southern Ethiopia. Dilla Town is located at 367 km to the south of Addis Ababa, the capital of the country, and 90 km from Hawassa, the center of the region, SNNPR. The study area has a longitude and latitude of 6°24′30″N 38°18′30″E Coordinates: 6°24′30″N 38°18′30″E, with an elevation of 1570 meters above sea level [22] and monthly temperature range of 10°c to 30°c [23].

### 2.2. Population

The source population for this study was all school adolescents from 10 to 19 years attending both private and government schools in Dilla Town, and the study population was adolescents attending the selected schools during the study period. Adolescents with known chronic diseases were excluded from the study.

### 2.3. Sample Size Determination

The sample size for this study was determined using two population proportion formulas considering prevalence of anemia among male and female taken from a previous cross-sectional study [24]. Accordingly, the sample size was determined using the following formula:

(1) $$\begin{array}{c} n=\frac{\left({\left\{Z_{\alpha /2}\sqrt{2pq}+Z\beta \sqrt{\left(p_{1}q_{1}\right)}+\sqrt{\left(p_{2}q_{2}\right)}\right\}}^{2}\right)}{\Delta^{2}}. \end{array}$$

In the formula, *n* = sample size, *Z*_*α*/2_ = at 95% CI which is 1.96, Power 80% is 0.84, *r* = 1 (ratio of exposed to nonexposed), *p*_1_ = percent of a male with anemia = 24.3% = 0.243, *p*_2_ = percent of a female with anemia = 18.1% = 0.181,

(2) $$\begin{array}{c} p=\frac{p1+p_{2}}{2}=0.212, \end{array}$$

(3) $$\begin{array}{c} q=1-p=0.788, \end{array}$$

(4) $$\begin{array}{c} q_{1}=1-p_{1}=0.757, \end{array}$$

(5) $$\begin{array}{c} q_{2}=1-p_{2}=0.819, \end{array}$$

(6) $$\begin{array}{c} \Delta =p_{1}-p_{2}-0.062. \end{array}$$

The calculated sample based on the above assumption was 688, and by adding ten percent nonresponse rate, the final sample size became 756 (378 males and 378 females).

### 2.4. Study Variable

#### 2.4.1. Dependent Variable

Adolescent anemia.

#### 2.4.2. Independent Variables

Demographic characteristics of the adolescents: age of adolescents, place of residence, grade of adolescents, educational status of mother and father, occupational status of mother and father, livening condition (with family or separate from family), parental condition (existence of mother and father), family size, and marital status.

Water and sanitation: source of drinking water, type of latrine, and wear shoe.

Dietary diversity/practice: food consumption, food frequency, food sources, postmeal consumption of tea and coffee, and school feeding program.

Knowledge of anemia: information on anemia, knowledge of food sources rich in iron, knowledge of causes of anemia, and knowledge of consequences of anemia.

Health and nutrition condition: body mass index for age, height for age, menstrual status, malaria endemicity, malaria and parasitic infection, deworming, and accessibility of adolescent health service.

### 2.5. Sampling Technique/Sampling Procedures

There are a total of 23 schools in Dilla Town out of which six schools were randomly selected with simple random sampling technique. Then, a total sample was proportionally allocated to each schools based on the number of adolescents in the selected schools. Finally, a simple random sampling (SRS) technique was applied to select study participants using the school roaster as a frame.

### 2.6. Data Collection Procedures and Measurements

For data collection, a pretested questionnaire, anthropometric assessment, haemoglobin measurement, and stool examination checklists were used. The questionnaire was adapted from different literature studies and EDHS [20, 24, 25]. For anthropometric assessment, height and weight were measured with standardized protocols and calibrated equipment. Weights were measured with minimal (light) clothing, shoes removed, and hats using a digital weighting scale (SECA, UNICEF, and Copenhagen) and recorded to the nearest 0.1 kg. Heights were measured using a wooden measuring board with a sliding head bar in Frankfurt position and recorded to the nearest 0.1 cm [26].

Haemoglobin determination was done for the selected students in the school compound by laboratory technicians that were working outside of the respective district. The haemoglobin concentration of each student was measured by taking a finger-prick blood sample using a HemoCue haemoglobinometer (HemoCue Hb 301+, Angelholm, Sweden). Standardization of the HemoCue haemoglobinometer was checked by crosschecking CBC machine [27]. Both interview and blood sample collection were taken from each adolescent student in a separate room. Haemoglobin measurements were adjusted for altitude, sex, and age as proposed by the WHO, and the cutoff point for anemia was based on the WHO recommendation for adolescents [2].

The stool examination was conducted by laboratory technicians using the portable microscope in each school compound. Stool samples were collected from each study participant using clean, wide-mouthed, and leak-proof stool cups and examined at the data collection site within 10–15 minutes of collection by the wet mount for identification of intestinal parasites.

The food and nutrition technical assistance questionnaire was used to collect data for dietary diversity. The types of food adolescents took within the last 24 hours were asked, and the information collected on dietary consumption was used to calculate the dietary diversity score (DDS).

Data were collected by four laboratory professionals and eight nurse diploma professionals. The data collectors were trained for two days on the data collection tools and anthropometric measurement procedures.

### 2.7. Data Analysis Procedures

Data were entered into EpiData version 3.1 and then exported to SPSS version 20 statistical packages for analysis. Anthropometric data were entered and calculated using the WHO AnthroPlus software. The dietary diversity scores (DDS) for 24-hour recall were calculated per adolescent by summing the number of different consumed food groups. The DDS was categorized into tertiles which include low dietary diversity with ≤2 food groups, medium dietary diversity with 3 food groups, and high dietary diversity with ≥4 food groups [28]. Stunting was defined using height for age *Z*-score of less than −2 SD. Descriptive statistics were computed to give a clear picture of background information and determine the prevalence. Then, data were analyzed using binary and multivariable logistic regression to assess the factors associated with anemia. Model fitness was done using the Hosmer–Lemeshow goodness of fit test for logistic regression. Statistical precision was estimated with 95% CI (confidence interval) and statistical significance determined at *p* value ≤0.05.

### 2.8. Data Quality Management

The questionnaire was originally developed in English and later translated into Amharic and Gedeofa. To keep the consistency of its content, the questionnaire was translated back to English by a language expert. Two-day training was given for data collectors and supervisors. Before data collection, a pretest was conducted among 38 students (5% of the sample size) to centexualize the questionnaires before the actual data collection and revisions were made accordingly. During the study period, the questionnaire was checked every evening for its completeness. Unrecorded data and unlikely responses were manually separated and reinterviewed the next day. All laboratory activities were performed by strictly following manufacturers' instructions and specific standard operating procedures. All anthropometric measurements were taken twice by two data collectors, and the mean values were used for data analysis. The weighing scale was calibrated with a known weight object regularly, and the scale indicator was checked against zero reading after weighing every adolescent.

## 3. Results

### 3.1. Sociodemographic and Socioeconomic Characteristics

From a total of 756 adolescent students expected to participate in this study, 742 (377 males and 365 females) were actually participated making the response rate 98.1%. Among the participants, about 50% (186) of males and about 40% (141) of females were in a late adolescence period with a mean age of 16 years (SD, 2.4) among males and 17 (SD, 2.1) among females. With regard to grade levels, 179 (47.5%) of males and 190 (52.1%) of females were from grade 5 to 8. Protestant religion followers constitute 293 (77.7%) among males and 237 (64.9%) among females in this study.

Regarding family characteristics, 179 (47.5) of male adolescents and 174 (47.7%) of female adolescents were from households with a family size of 5–7 members. With respect to family education, 191 (50.7) of males' fathers and 198 (54.2) of females' fathers had attended secondary school or above (Table 1).

### 3.2. Dietary Practices and Related Factors

Information about the source of food shows that the majority of the family of male (70.8%) and female (76.4%) adolescents achieve food need through buying/purchasing. Most of the study participants in both sexes 272 (72.1%) among male and 262 (71.8%) among female had low DDS in the last 24-hour recall. Concerning daily meal frequency, 335 (88.9%) of male adolescents and 342 (93.7%) female adolescents eat three times or more (Table 2).

### 3.3. Health Services, Personal Practices, and Related Factors

About 50% (140) of males and 43.2% (115) of female respondents have poor knowledge about anemia. About 10.3% (39) of male adolescents and 9.6% (35) of female adolescents have history of malaria infection in the last one month. Regarding stool parasite assessment result, 104 (31.5%) of male study participants and 78 (22.0%) of female adolescents have parasite infestation (Table 3).

### 3.4. Prevalence of Anemia and Anthropometric Measurements

In this study, the overall prevalence of anemia was 157 (21.1%) and it is 85 (22.5%) among male adolescents and 72 (19.7%) among female adolescents. The mean haemoglobin levels were 14.4 (±2.3) among male and 13.4 (±1.9) among female study participants. Regarding nutritional status of the adolescents, the level of stunting was 48 (12.7%) among males and 37 (10.1%) among females. According to BMI *Z*-score for age result, 39 (10.3%) of male adolescents and 19 (5.2%) of female adolescents were thin (Table 4).

### 3.5. Factors Associated with Anemia among School Adolescents in Dilla Town

The multivariable analysis result showed that age category, the frequency of eating fibre foods, and presence of stool parasites were significantly associated with the prevalence of anemia among male adolescent students and malaria endemicity and presence of stool parasites were significantly associated with the prevalence of anemia among female adolescent students.

With regard to factors associated with anemia among males, adolescents in the age range of 10–13 years were 76% less likely to develop anemia (AOR 0.24, 95% CI, 0.07–0.77) compared to those respondents in the age group of 17–19 years. Adolescent males who eat fibre foods every day were 88% less likely to develop anemia compared to those who eat none through the week days (AOR 0.12, 0.02–0.64). Adolescent males who have no stool parasite were 99.5% less likely to develop anemia than adolescent males who have stool parasite (AOR 0.05, 95% CI, 0.02–0.09).

Adolescent females from malaria endemic area were 2.5 times more likely to develop anemia than adolescent females from the less endemic area (AOR 2.52, 95% CI, 1.12–5.62). Adolescent females who have no intestinal parasite were 99.5% less likely to develop anemia than adolescent females who have intestinal parasite (AOR 0.05, 95% CI, 0.01–0.11) (Table 5).

## 4. Discussion

The result of this study indicated that the overall prevalence of anemia among adolescents in the study areas was 21.1%. According to the WHO established criterion, this prevalence is of a moderate public health concern. The comparative assessment revealed that male adolescents (22.5%) were slightly more anemic than female (19.3%) adolescents. However, the difference was found to be not statistically significant. Another finding of a study conducted among adolescents in Wonago District, Gedeo Zone, Southern Ethiopia, found significantly higher prevalence of anemia among male adolescents (24.3%) than among female adolescents (18.1%) [24]. The finding from South Kerala in India also found that male adolescents were more anemic than female adolescents [29]. However, the finding of the study conducted in Bonga Town, Southwest Ethiopia, found that the prevalence of anemia among male adolescents was lower among males (9.4%) than females (19.3%) [25]. This might be due to certain difference in the population included in the study conducted in Bonga Town where the study included older adolescents, and the higher prevalence of anemia among females might be due to mensus. The finding of this study in this regard implies that the trend of considering male adolescents as a less vulnerable group to anemia than the female counterparts in programs attempting to intervene nutritional problems such as anemia should be reconsidered.

Regarding the overall prevalence, the result of this study revealed that the prevalence of anemia was comparable to the other studies in Ethiopia including those conducted in Wonago Town [24] and Bonga Town [25]. On the other hand, the overall prevalence is much lower than the study conducted in India (62.0% in females and 46.1% in males, respectively) [29] and Chennai, Tamil Nadu, among adolescent female students which found 78.8% [30]. The difference in the finding of our study from different regions of the world could be due to the difference in cultural and behavioural practices in different areas of the world. This could also be due to Ethiopia being the country with widespread teff, cereal rich in iron, consuming country than other developing countries.

In the present study, the prevalence of anemia among adolescent males whose age 10–13 years is lower than the prevalence among adolescent males with an age group of 17–19 years. This study is not in agreement with another comparable study conducted in Wonago District, Gedeo Zone, which indicated anemia to be higher among early adolescent periods (10–13 years) compared to the late adolescent period (17–19 years) [24]. The differences may be due to the larger number of menstruating adolescents (females) in this study compared to the previous one.

This study also revealed that adolescent males who eat fibre foods every day were less likely to develop anemia than adolescent males who eat none throughout the weeks. This finding is in line with the study done in Alexandria, Egypt, which reported high consumption of whole wheat bread and bread, low dietary intake of iron-rich foods, and low consumption of vitamin C rich foods to have a significant association with anemia [31]. This suggests that dietary iron intake in fruits and other vegetables is good sources of Vit-A and Vit-C and those food items are also good iron absorption enhancers [32]. Adolescent males who have no intestinal parasite were less likely to develop anemia than adolescent males who have intestinal parasites. This finding is also in agreement with the finding reported from Bonga Town, Southwest Ethiopia, which indicated intestinal parasite infection as one of the factors increasing the risk of anemia among adolescents [25]. This might be due to the fact that most identified intestinal parasites have their own contribution to blood loss and/or red cell destruction [33].

Regarding the factors associated with anemia among adolescent females, those coming from where malaria was common in their area were more likely to develop anemia than adolescent females for whom malaria is not common in their area of residence. This is because malaria is a main cause of anemia in adolescents in Sub-Saharan Africa, and it behaves such as an endemic disease in some areas, such as Ethiopia. Malaria infection is associated with a reduction in haemoglobin levels by the destruction of red blood cells, frequently leading to anemia. This study also identified that the prevalence of anemia among female adolescents who had been infected with intestinal parasites was significantly higher compared to those not infected with intestinal parasites. A similar finding was reported from a study conducted in Siaya District, Kenya; female adolescents who were infected by worms were to develop anemia as opposed to those who did not have worm infestation [34]. This finding is also in contrary to a similar study conducted in Tigray, Northern Ethiopia [35]. This implies that intestinal parasites have also their own contribution for developing anemia among females.

The current study attemted to reveal the level of anemia among male and female adolescents employing a comparative cross-sectional study to have enough sample among the comparison groups. It also addressed a wide range of factors that are associated with anemia in both sexes. The measurement used to assess anemia using HemoCue Hb 301+machine is precise and accurate proxy indicator for anemia study. The limitation of this study was the exclusion of adolescents who are out of school. In addition, there could also be a social-desirability bias in dietary questions asked for food habit.

## 5. Conclusion

The prevalence of anemia was higher in male adolescent students than females. Age, the frequency of eating fibre foods, and intestinal parasites were significantly associated factors for anemia among male adolescents, and malaria endemicity and stool parasites were factors significantly associated with anemia among female adolescent students. Hence, nutrition programs focusing on anemia among adolescents should give due consideration for both sexes and male adolescents need to have more emphasis. Furthermore, stronger studies with better designs need to be conducted to confirm this finding and to come up with concrete evidence for policy making.

## Figures and Tables

**Table 1:** Sociodemographic and socioeconomic characteristics of male and female adolescent students in Dilla Town, Gedeo Zone, Southern Ethiopia, May 2018.

Variables	Male number (%)	Female number (%)
Age category	377	365
Early adolescence (10–13 yrs)	61 (16.2)	74 (20.3)
Middle adolescence (14–16 yrs)	130 (34.5)	150 (41.1)
Late adolescence (17–19 yrs)	186 (49.3)	141 (38.6)
Mean age (±SD)	16 ± 2.4	17 ± 2.1

Grade	377	365
5–8	179 (47.5)	190 (52.1)
9-10	140 (37.1)	142 (38.9)
11-12	58 (15.4)	33 (9.0).

Ethnicity	377	365
Gedeo	258 (70.9)	214 (61.7)
Oromo	44 (12.1)	41 (11.8)
Amhara	31 (8.5)	36 (10.4)
Gurage	25 (6.9)	43 (12.4)
Others^*a*^	19 (5.0)	31 (8.6)

Religion	377	365
Protestant	293 (77.7)	237 (64.9)
Orthodox	67 (17.8)	95 (26.0)
Muslim	7 (1.9)	21 (5.8)
Catholic	10 (2.7)	12 (3.3)

Father's educational status	377	365
Illiterate/cannot read or write	26 (6.9)	13 (3.6)
Can read and write	91 (24.1)	82 (22.5)
Primary school	69 (18.3)	72 (19.7)
Secondary school and above	191 (50.7)	198 (54.2)

Mother's educational status	377	365
Illiterate/cannot read or write	65 (17.2)	42 (11.5)
Can read and write	92 (24.4)	98 (26.8)
Primary school	112 (29.7)	101 (27.7)
Secondary school and above	108 (28.6)	124 (34.0)

Father's occupational status	377	365
Farmer	73 (19.8)	54 (15.3)
Government employee	155 (42.1)	147 (41.8)
Merchant	140 (38.0)	151 (42.9)
Others^*b*^	9 (2.4)	13 (3.6)

Mother's occupational status	377	365
Housewife	152 (41.1)	156 (43.9)
Farmer	21 (5.7)	14 (3.9)
Government employee	80 (21.6)	71 (20.0)
Merchant	177 (31.6)	114 (32.1)
Others^*c*^	7 (1.9)	10 (2.7)

Your current marital status	377	365
Never married	341 (90.5)	337 (92.3)
On promise	25 (6.6)	16 (4.4)
Married	11 (2.9)	12 (3.3)

Family size	377	365
≤4	35 (9.3)	33 (9.0)
5–7	179 (47.5)	174 (47.7)
≥8	163 (43.2)	158 (43.3)

Place of residence	377	365
Urban	329 (87.3)	337 (92.3)
Rural	48 (12.7)	28 (7.7)
Parental status	377	365
Both parent alive	343 (91.0)	333 (91.2)
Father alive	7 (1.9)	6 (1.6)
Mother alive	23 (6.1)	25 (6.8)
Both parent died	4 (1.1)	1 (0.3)

Current living status	377	365
Living with parent	305 (80.9)	316 (86.6)
Living with relatives	18 (4.8)	26 (7.1)
Living separately from family	54 (14.3)	23 (6.3)

^*a*^ = Wolaita, Silte, Sidama, and others; ^*b*^ = private employee, daily laborer, student, and others; ^*c*^ = private employee, daily laborer, and student.

**Table 2:** Dietary practices and related factors among male and female adolescent students in Dilla Town, Gedeo Zone, Southern Ethiopia, May 2018.

Variables	Male (*n* = 377) number (%)	Female (*n* = 365) number (%)
Daily meal frequency (usual)	377	365
Two times or below	42 (11.1)	23 (6.3)
Three times or more	335 (88.9)	342 (93.7)

Daily meal frequency (24 hours)	377	365
Two times or below	113 (30.0)	83 (22.7)
Three times or more	264 (70.0)	282 (77.3)

Main sources of family food needs	377	365
Grow their own	99 (26.3)	74 (20.3)
Buy/purchase	267 (70.8)	279 (76.4)
Subsidies/food aid	11 (2.9)	12 (3.3)

DDS in last 24 hours	377	365
Low	272 (72.1)	262 (71.8)
Medium	100 (26.5)	99 (27.1)
High	5 (1.3)	4 (1.1)

Green leafy vegetable consumption	377	365
None	15 (4.0)	20 (5.5)
Once a week	53 (14.1)	32 (8.8)
Twice in a week	98 (26.0)	99 (27.1)
Every other day	80 (21.2)	74 (20.3)
Every day	87 (23.1)	86 (23.6)
Do not remember	44 (11.7)	54 (14.8)

Frequency of fibre foods	377	365
None	24 (6.4)	29 (7.9)
Once a week	83 (22.0)	61 (16.7)
Twice in a week	71 (18.8)	81 (22.2)
Every other day	61 (16.2)	47 (12.9)
Every day	67 (17.8)	81 (22.2)
Do not remember	71(18.8)	66 (18.1)

Frequency of foods of animal origin	377	365
None	34 (9.0)	21 (5.8)
Once a week	147 (39.0)	118 (32.3)
Twice in a week	75 (19.9)	105 (28.8)
Every other day	38 (10.1)	48 (13.2)
Every day	42 (11.1)	41 (11.2)
Do not remember	41 (10.9)	32 (8.8)

Consumption of tea after a meal	377	365
Always	118 (31.3)	123 (33.7)
Sometimes	207 (54.9)	194 (53.2)
Not at all	40 (10.6)	33 (9.0)
Do not remember	12 (3.2)	15 (4.1)

**Table 3:** Health service, personal practices, and related factors among male and female adolescent students in Dilla Town, Gedeo Zone, Southern Ethiopia, May 2018.

Variables	Male (*n* = 377) number (%)	Female (*n* = 365) number (%)
Knowledge of anemia	281	266
Good knowledge	141 (50.2)	151 (56.8)
Poor knowledge	140 (49.8)	115 (43.2)

Malaria endemicity	377	365
Yes	184 (48.8)	178 (48.8)
No	193 (51.2)	187 (51.2)

History of malaria last one month	377	365
Yes	39 (10.3)	35 (9.6)
No	338 (89.7)	330 (90.4)

Deworming in the last one month	377	365
Yes	48 (12.7)	62 (17.0)
No	329 (87.3)	303 (83.0)

Menstruation		365
Yes		329 (90.1)
No		36 (9.9)

Days of menstruation		329
<3 days		183 (55.6)
>3 days		146 (44.4)

Frequency of pad change		329
<3 times		271 (82.4)
>3 times		58 (17.6)

Source of drinking water	377	365
Piped water	295 (78.2)	318 (87.1)
Stand pipe	31 (8.2)	20 (5.5)
Protected spring	51 (13.5)	27 (7.4)

Shoe wearing frequency	377	365
Some of the time	90 (23.9)	35 (9.6)
Most of the time	87 (23.1)	44 (12.1)
Always	200 (53.1)	286 (78.4)

**Table 4:** Prevalence of anemia among male and female adolescents in Dilla Town, Southern Ethiopia, May 2018.

Variables	Male (*n* = 377)	Female (*n* = 365)
	Number (%)	Number (%)
Anemia status	377	365
Anemic	85 (22.5)	72 (19.7)
Nonanemic	292 (77.5)	293 (80.3)

Types of anemia	85	72
Sever	1 (0.3)	4 (1.1)
Moderate	46 (12.2)	43 (11.8)
Mild	38 (10.1)	25 (6.8)
Mean (SD)	14.4 ± 2.3	13.4 ± 1.9

HAZ	377	365
Stunted	48 (12.7)	37 (10.1)
Normal	329 (87.3)	328 (89.9)

BAZ	377	365
Thin	39 (10.3)	19 (5.2)
Normal	338 (89.7)	346 (94.8)

**Table 5:** Associated factors of anemia among male and female adolescent students in Dilla Town, Southern Ethiopia, May 2018.

Variables	Male				Female			
	Anemic	Nonanemic	COR (95% CI)	AOR (95% CI)	Anemic	Nonanemic	COR (95% CI)	AOR (95% CI)
Age	85	292			72	293		
10–13	25 (41.0)	36 (59.0)	0.29 (0.15–0.55)^*∗*^	0.24 (0.07–0.77)^*∗*^	29 (39.2)	45 (61.8)	0.27 (0.14–0.52)^*∗*^	0.27 (0.05–1.38)
13–16	29 (22.3)	101 (77.7)	0.69 (0.39–1.23)	0.44 (0.17–1.11)	22 (14.7)	128 (85.3)	1.02 (0.53–1.95)	0.90 (0.30–2.66)
17–19	31 (16.7)	155 (83.3)	1	1	21 (14.9)	120 (85.1)	1	1

Grade	85	292			72	293		
5–8	51 (28.5)	128 (71.5)	1	1	46 (24.2)	144 (75.8)	1	1
9-10	24 (17.1)	116 (82.9)	1.93 (1.12–3.33)^*∗*^	0.62 (0.24–1.58)	21 (14.8)	121 (85.2)	1.84 (1.04–3.25)^*∗*^	1.63 (0.57–4.63)
11-12	10 (17.2)	48 (82.8)	1.91 (0.89–4.07)	0.56 (0.14–2.10)	5 (15.2)	28 (84.8)	1.79 (0.65–4.90)	0.84 (0.14–4.79)

Father's education	85	292			72	293		
No formal education	8 (30.8)	18 (69.2)	0.73 (0.30–1.79)		3 (23.1)	10 (76.9)	0.64 (0.17–2.46)	0.39 (0.06–2.47)
Read and write	14 (15.4)	77 (84.6)	1.79 (0.93–3.47)		22 (26.8)	60 (73.2)	0.53 (0.28–0.97)^*∗*^	0.57 (0.18–1.73)
Primary school	16 (23.2)	53 (76.8)	1.08 (0.56–2.07)		15 (20.8)	57 (79.2)	0.73 (0.37–1.45)	0.89 (0.27–2.91)
Secondary and above	47 (24.6)	144 (75.4)	1		32 (16.2)	166 (83.8)	1	1

Father's occupation	85	292			72	293		
Farmer	13 (17.8)	60 (82.2)	1		7 (13.0)	47 (87.0)	1	1
Government employee	35 (22.6)	120 (76.4)	0.74 (0.36–1.50)		21 (14.3)	126 (85.7)	0.89 (0.35–2.23)	2.00 (0.42–9.53)
Merchant	36 (25.7)	104 (74.3)	0.63 (0.31–1.27)		43 (28.5)	108 (71.5)	0.37 (0.15–0.89)^*∗*^	1.38 (0.36–5.20)
Others	1 (11.1)	8 (88.9)	0.80 (0.57–1.12)		1 (7.7)	12 (92.3)	0.53 (0.35–0.80)^*∗*^	0.26 (0.01–4.16)

Mother's occupation	85	292			72	292		
Housewife	37 (24.3)	115 (75.7)	1		26(16.7)	130 (83.3)	1	1
Farmer	4 (19.1)	17 (80.9)	1.37 (0.43–4.32)		1 (7.1)	13 (92.9)	2.60(0.32–20.75)	1.31 (0.11–15.72)
Government employee	17 (21.3)	63 (78.7)	1.19 (0.62–2.29)		11 (15.5)	60 (84.5)	1.09 (0.51–2.35)	0.79 (0.25–2.47)
Merchant	24 (20.5)	93 (79.5)	1.25 (0.69–2.23)		33 (29.8)	80 (70.2)	0.47 (0.26–0.84)^*∗*^	0.68 (0.27–1.69)
Others	3 (42.9)	4 (57.1)	1.08 (0.89–1.29)		1 (10.0)	9 (90.0)	0.73 (0.59–0.87)^*∗*^	1.29 (0.14–11.19)

Shoe wearing	85	292			72	293		
Some of the time	17 (18.9)	73 (81.1)	1.47 (0.79–2.72)		6 (17.1)	29 (82.9)	0.95 (0.37–2.42)	1.19 (0.30–4.79)
Most of the time	17 (19.5)	70 (80.5)	1.41 (0.76–2.61)		19 (43.2)	25 (56.8)	0.26 (0.13–0.50)^*∗*^	0.39 (0.12–1.27)
Always	51 (25.5)	149 (84.5)	1		47 (16.4)	239 (83.6)	1	1

Sources of family food	85	292			72	293		
Grow their own	18 (18.2)	81 (81.8)	1		8 (10.8)	66 (89.2)	1	1
Buy/purchase	65 (24.3)	202 (75.7)	0.69 (0.38–1.23)		62 (22.2)	217 (77.8)	0.42 (0.19–0.93)^*∗*^	0.55 (0.17–1.67)
Subsidies/food aid	2 (18.2)	9 (81.8)	1.00 (0.19–5.02)		2 (16.7)	10 (83.3)	0.61 (0.11–3.27)	5.85 (0.33–101.37)
Fibre foods per week	85	292			72	293		
None	4 (16.7)	20 (83.3)	1	1	4 (13.8)	25 (86.2)	1	1
Once a week	11 (13.3)	72 (86.7)	1.31 (0.37–4.55)	0.68 (0.12–3.67)	11 (18.0)	50 (82.0)	0.73 (0.21–2.52)	0.53 (0.09–2.94)
Twice in a week	18 (25.4)	53 (74.6)	0.59 (0.17–1.95)	0.24(0.04–1.27)	8 (9.9)	73 (90.1)	1.460 (0.40–5.26)	3.36 (0.50–22.24)
Every other day	11 (18.0)	50 (82.0)	0.91 (0.26–3.19)	0.61(0.11–3.36)	12 (25.5)	35 (74.5)	0.47 (0.13–1.61)	0.73 (0.13–4.12)
Every day	27 (40.3)	40 (59.7)	0.29 (0.09–0.96)^*∗*^	0.12 (0.02–0.64)^*∗*^	28 (34.6)	53 (65.4)	0.30 (0.09–0.95)^*∗*^	0.63 (0.12–3.11)
Do not remember	14 (19.7)	57 (80.3)	0.81 (0.24–2.76)	0.55 (0.10–2.95)	9 (13.6)	57 (86.4)	1.01 (0.28–3.60)	1.55 (0.26–9.06)

Animal food per week	85	292			72	293		
None	3 (9.1)	31 (90.9)	1	1	6 (28.6)	15 (71.4)	1	
Once a week	38 (25.9)	109 (84.1)	0.28 (0.08–0.96)^*∗*^	0.36 (0.08–1.58)	20 (16.9)	98 (73.1)	1.96 (0.67–5.66)	
Twice in a week	16 (21.3)	59 (78.7)	0.36 (0.09–1.32)	0.43 (0.09–2.09)	20 (19.0)	85 (81.0)	1.70 (0.58–4.93)	
Every other day	8 (21.1)	30 (78.9)	0.36 (0.08–1.49)	0.64 (0.11–3.61)	9 (18.8)	39 (81.2)	1.73 (0.52–5.71)	
Every day	11 (26.2)	31 (73.8)	0.27 (0.06–1.07)	0.74 (0.13–4.01)	11 (26.8)	30 (73.2)	1.09 (0.33–3.52)	
Do not remember	9 (22.0)	32 (78.0)	0.34 (0.08–1.39)	0.40 (0.07–2.23)	6 (18.8)	26 (81.2)	1.73 (0.47–6.34)	

Stool parasite	82	272			63	267		
No	30 (10.9)	246 (89.1)	0.06 (0.03–0.11)^*∗*^	0.05 (0.02–0.09)^*∗*^	13 (5.8)	213 (94.2)	0.07 (0.03–0.13)^*∗*^	0.05 (0.01–0.11)^*∗*^
Yes	52 (66.7)	26 (33.3)	1	1	50 (48.1)	54 (51.9)	1	1

Malaria endemicity	85	292			67	293		
Yes	37 (20.1)	147 (79.9)	1.31 (0.80–2.13)		27 (15.2)	151 (84.8)	1.77 (1.04–3.00)^*∗*^	2.52 (1.12–5.62)^*∗*^
No	48 (24.9)	145 (75.1)	1		45 (24.1)	142 (75.9)	1	1

Start menstruating					72	293		
Yes					57 (17.3)	272 (82.7)	1	1
No					15 (41.7)	21 (58.3)	0.29 (0.14–0.60)^*∗*^	2.56 (0.53–12.28)

^*∗*^Statistically significant at *p* < 0.05; COR = crudes odds ratio; AOR = adjusted odds ratio.

## Data Availability

The data underlying this study are readily available and can be accessed as needed from the corresponding author.
